# Adding Papillomacular Bundle Measurements to Standard Optical Coherence Tomography Does Not Increase Sensitivity to Detect Prior Optic Neuritis in Patients with Multiple Sclerosis

**DOI:** 10.1371/journal.pone.0155322

**Published:** 2016-05-12

**Authors:** Mona Laible, Sven Jarius, Friedericke Mackensen, Annette Schmidt-Bacher, Michael Platten, Jürgen Haas, Philipp Albrecht, Brigitte Wildemann

**Affiliations:** 1 Department of Neurology, Heidelberg University Hospital, Heidelberg, Germany; 2 Department of Ophthalmology, Heidelberg University Hospital, Heidelberg, Germany; 3 Department of Ophthalmology, St. Vincentius-Kliniken gAG, Karlsruhe, Germany; 4 Department of Neurology, Medical Faculty, Heinrich Heine University Düsseldorf, Germany; Julius-Maximilians-Universität Würzburg, GERMANY

## Abstract

**Purpose:**

To improve the detection of retinal nerve fiber layer (RNFL) thinning in multiple sclerosis (MS), a special peripapillary ring scanning algorithm (N-site RNFL, N-RNFL) was developed for spectral domain optical coherence tomography (SD-OCT). In contrast to the standard protocol (ST-RNFL) scanning starts nasally, not temporally, and provides an additional sector of analysis, the papillomacular bundle (PMB). We aimed to ascertain whether the temporal RNFL differs between the two techniques, whether N-RNFL is more sensitive than ST-RNFL to detect previous optic neuritis (ON), and whether analyzing the PMB adds additional sensitivity. Furthermore, we investigated whether RNFL is associated with disease severity and/or disease duration.

**Methods:**

We conducted a cross-sectional case-control study of 38 patients with MS, of whom 24 had a history of ON, and 40 healthy controls (HC). Subjects with ON within the previous 6 months were excluded. Records included clinical characteristics, visual evoked potentials (VEP), and SD-OCT in both techniques.

**Results:**

In a total of 73 evaluable MS eyes, temporal N-RNFL was abnormal in 17.8%, temporal ST-RNFL in 19.2%, and the PMB-RNFL in 21.9%. In ON eyes, the sensitivity of temporal N-RNFL and ST-RNFL did not differ significantly (37.0%/33.3%, p = 0.556). The sensitivity of VEP was 85.2%. RNFL thickness was associated with disease severity in all eyes, with and without a history of ON, and with disease duration.

**Conclusion:**

The two OCT techniques detected previous ON with similar sensitivity, but the sensitivity of VEPs was superior to that of both N-RNFL and ST-RNFL. Our results indicate that the widely used ST-RNFL technique is appropriate for peripapillary RNFL measurements in MS patients.

## Introduction

Optical coherence tomography (OCT) allows precise measurement of retinal nerve fiber layer (RNFL) thickness and is thus a promising tool for the detection of prior optic neuritis (ON). Numerous studies have used OCT to detect and characterize thinning of the peripapillary RNFL in the context of multiple sclerosis (MS) [1–5], clinically isolated syndrome suggestive of MS (CIS) [6], or as an isolated syndrome [7].

Good inter-method agreement of Spectralis OCT and Cirrus HD-OCT segmentation techniques has been reported [8], but less is known about the comparability of the two available circle scan protocols of the Spectralis SD-OCT device. The standard RNFL scanning algorithm applied in ophthalmology (ST-RNFL) consists of a 12° peripapillary ring scan which starts scanning in the temporal sector and provides post-processing analysis to distinguish six peripapillary sectors. For RNFL measurements in neurological disorders like MS, the N-site software package was developed. This provides a 12° ring scanning algorithm (N-RNFL) which begins scanning in the nasal sector and provides the output of seven sectors, including the papillomacular bundle (PMB) for post-acquisition analysis. The hypothesis was that continuously scanning over the temporal quadrant and adding an additional area of measurement for the papillomacular bundle might decrease the risk of artifacts and provide additional sensitivity, respectively. Since the PMB, which conveys information from the fovea, i.e. the central macular structure mainly responsible for detailed visual and color functions, is predominantly affected in ON, we hypothesized that the sensitivity of N-RNFL to detect previous ON would exceed that of ST-RNFL. The aim of this study was to investigate the agreement of the two Spectralis OCT circle scan protocols in a subset of MS patients and in a group of healthy controls (HC). We attempted to address whether absolute values of RNFL thickness differed globally or in distinct scan sectors between N-RNFL and ST-RNFL. Furthermore, we aimed to determine the sensitivity and specificity of both methods for previous ON in comparison to the established diagnostic standard, full-field visual evoked potentials (VEP).

## Materials and Methods

### Patients

The study protocol was approved by the ethics committee of the University of Heidelberg, Germany (statement S-310/2011) and the study was conducted in accordance with the Declaration of Helsinki (1964) in its currently applicable version, the guidelines of the International Conference on Harmonization of Good Clinical Practice (ICH-GCP), and applicable German laws. All participants gave written informed consent. We recruited participants between March 2011 and June 2012. Data were collected from 44 consecutive patients referred to our neurology department for routine assessment.

The inclusion criteria were (a) a diagnosis of MS according to the McDonald criteria of 2005 [9], (b) age at least 18 years, (c) absence of ON in the previous 6 months (based on previous recommendations from OCT trials of ON [10]), (d) absence of competing ocular pathologies, and (e) refraction values between -5 and +5 diopters.

Six patients had to be excluded because their refraction values were below or above the predetermined limits, OCT was not feasible, or scan quality was insufficient [11]. Based on the OSCAR IB criteria, we excluded four RNFL measurements post hoc (insufficient fundus illumination n = 2, wrongly centered ring scan n = 1, papillary edema as visible retinal pathology n = 1) [11]. A diagnosis of previous ON was established for all eyes separately based on a clinical history of ON as assessed by interview and scrutiny of patient records. We reviewed our local database for (VEP) recordings, and only VEP conducted after the last episode of ON were considered. VEP to 50 arc minutes pattern reversal (3 Hz) achromatic checks were recorded according to standard procedures over Oz (midline occipital electrode) of the international 10–20 system, with the reference being Cz in agreement with established recommendations [12]. We used an Alpine Biomed device with the software Dantec keypoint.net, version 2.02 (Alpine Biomed Corp., Fountain Valley, CA, USA). VEP amplitudes and latencies were compared with the normative data available at our laboratory and rated as pathologic or normal.

For all patients, the following data were recorded: Corrected visual acuity (VA), RNFL thickness measured with both OCT scanning protocols as global values (ST-RNFL-G and N-RNFL-G) and within the different anatomical peripapillary sectors, and Expanded Disability Status Scale (EDSS). VEP recordings were available from 73 MS eyes.

### Healthy controls

The 40 HC were age-matched volunteers who were not financially compensated for participating in the study.

### Optical coherence tomography

All participants underwent SD-OCT examination using the Heidelberg Engineering Spectralis SD-OCT device (Heidelberg Engineering, Heidelberg, Germany). For both eyes of each participant, the peripapillary RNFL thickness was assessed using the 12° circle scan employing the eye tracker system (TrueTrack^®^; Heidelberg Engineering). All peripapillary circle scans centered on the optic disk were performed using both scanning algorithms, ST-RNFL (retina menu) and N-RNFL (N-site/axonal menu). While for the ST-RNFL algorithm scanning begins and ends in the temporal sector, the N-RNFL algorithm starts and ends nasally. All scans were obtained at 100 ART in the high-resolution setting, the peripapillary ring centered on the disk in accordance with the OSCAR-IB criteria [12]. Three independent peripapillary ring scans were performed with each algorithm. Data from six peripapillary sectors were obtained with the ST-RNFL scan (c.f. Table 1, S1 Dataset), while the N-RNFL technique adds a seventh sector, the PMB [13]. One run of three circle scans was conducted with the ST-RNFL mode, one with the N-RNFL mode. Later, the median value of each set of three scans was calculated.

**Table 1:** RNFL thickness values in controls and MS-patients in all peripapillary sectors and comparison among groups.

	Thickness, μm, mean value, SD			p-values		
	Controls (n = 74)	Non-ON- eyes (n = 49)	ON-eyes (n = 24)	Non-ON-eyes versus controls	Non-ON versus ON-eyes	ON-eyes versus controls
**N-RNFL**						
N-RNFL-G	99.4±8.6	98.3±14.2	87.3±31.7	**p<0.002**	**p = 0.010**	**p = 0.002**
N-RNFL-PMB	56.9±7.71	51.1±11.7	43.9±12.9	**p<0.002**	**p = 0.034**	**p = 0.002**
N-RNFL-T	74.6±11.2	66.5±14.6	57.4±17.8	**p<0.002**	**p = 0.028**	**p = 0.004**
N-RNFL-TS	136.1±13.2	135.3±26.7	121.2±26.5	**p<0.002**	p = 0.064	**p = 0.016**
N-RNFL-TI	146.8±18.9	139.4±29.0	139.7±30.1	**p<0.002**	p = 0.092	**p = 0.008**
N-RNFL-N	74.6±14.4	74.4±17.4	75.3±33.7	p = 0.102	p = 0.140	**p = 0.032**
N-RNFL-NS	106.5±23.4	107.1±25.6	100.9±38.2	**p = 0.006**	**p = 0.022**	**p = 0.004**
N-RNFL-NI	108.1±20.9	113.3±24.5	102.8±30.5	**p = 0.002**	**p = 0.014**	**p = 0.006**
**ST-RNFL**						
ST-RNFL-G	99.4±7.2	94.0±14.2	87.8±17.8	**p<0.002**	**p = 0.030**	**p = 0.004**
ST-RNFL-T	73.9±11.3	67.1±16.6	58.2±21.6	**p<0.002**	**p = 0.026**	**p = 0.010**
ST-RNFL-TS	136.6±12.6	132.5±27.9	121.1±21.0	**p = 0.012**	p = 0.228	p = 0.148
ST-RNFL-TI	145.9±18.6	140.7±29.1	127.0±23.1	**p = 0.002**	p = 0.098	**p = 0.022**
ST-RNFL-N	74.3±13.5	72.8±14.9	67.0±22.6	p = 0.108	p = 0.252	**p = 0.034**
ST-RNFL-NS	107.5±23.6	108.7±19.9	99.4±42.5	**p = 0.048**	p = 0.104	**p = 0.014**
ST-RNFL-NI	105.3±19.9	108.2±23.1	92.0±29.5	**p = 0.010**	p = 0.082	**p = 0.024**

Abbreviations: RNFL = retinal nerve fibre layer, N-RNFL = peripapillary scan technique with additional examination of the papillomacular bundle, ST-RNFL = standard peripapillary scan technique, SD = standard deviation. G = global, PMB = papillo-macular bundle, T = temporal sector, TS = temporal superior sector, TI = temporal inferior sector, N nasal sector, NS = nasal superior sector, NI = nasal inferior sector. p-values below the level of significance of 0.05 are bold.

Normative data have been obtained by the manufacturer of the SD-OCT for both scanning algorithms. The RNFL thickness threshold value was based on the manufacturer’s reference values provided in the built-in, age-adjusted OCT database, where values no more than two standard deviations above or below the overall mean for the age-matched internal controls are considered within normal limits.

All scans were performed by an experienced operator (M.L.) and were reviewed for sufficient signal strength, correct centering, and beam placement as well as segmentation (F.M., A.S.) according to the OSCAR-IB criteria [11]. Corrected VA and refraction were measured on a decimal scale (0.1–1.0) at the time of OCT examinations using a NIDEK auto-refractometer AR 660A, NIDEK Co., Gamagori, Japan.

### Statistical analysis

Statistical analyses were performed using SPSS (version 21.0 for Windows; SPSS Inc., Chicago, IL, USA).We applied generalized estimation equation (GEE) models accounting for within-subject inter-eye correlations using an exchangeable correlation structure correcting for age and sex to explore differences of OCT measures between MS patients (ON eyes, non-ON eyes) and healthy controls. A Bonferroni correction was used to adjust for comparison of multiple groups. A GEE regression analysis was used to test for associations between OCT measures in MS eyes and VA, EDSS, and disease duration of MS. The McNemar test was used for comparison of dichotomous proportions between the different methods of measurement.

Depending on the scale level of the variables, we used the Mann-Whitney U test for continuous, but not normally distributed variables and the chi-squared test for categorical variables to explore differences between the MS and HC groups. The one-sample Kolmogorov-Smirnov Test was used to test for normal distribution.

The level of significance was set at 0.05. A Bonferroni correction was used to correct for multiple comparisons Statistical tests, including the uni- and multivariate models and the p values they yield can only be interpreted descriptively.

## Results

### Patient group

The mean age of the 38 patients included was 36.3±12.3 years, mean disease duration was 6.5±6.3 years (minimum 1 month, maximum 23 years), and median EDSS was 2.0 (interquartile range, IQR, 1.5–4.0). Twenty-five of the 38 patients were female (65.8%). Thirty-two subjects had relapsing-remitting MS, four had secondary progressive MS, and two had primary progressive MS. A total of 73 eyes of MS patients were studied with both techniques. A history of ON was found in 24 eyes. In five subjects the ON had been bilateral.

### Control group

The mean age of the 40 age- and sex-matched HC was 29.45±8.6 years, and 28 of them (70.0%) were female. The gender distribution was similar in the patient group and the HC group (Chi-square test p = 0.894), as was the age pattern (Mann-Whitney U test p = 0.078). High-quality OCT measurements were obtained from 74 eyes.

### Visual function

The median corrected VA was 0.8 (IQR 0.8–1.0) in the patient group and 1.0 (IQR 0.8–1.0) in the HC group. In ON eyes the median corrected VA was 0.8 (IQR 0.6–0.9), and in eyes without history of ON it was 1.0 (IQR 0.8–1.0). Ten eyes had a corrected VA of 0.5 or below (13.7%).

### Differences in RNFL thickness in ON eyes, non-ON eyes and healthy controls

**Fig 1:**
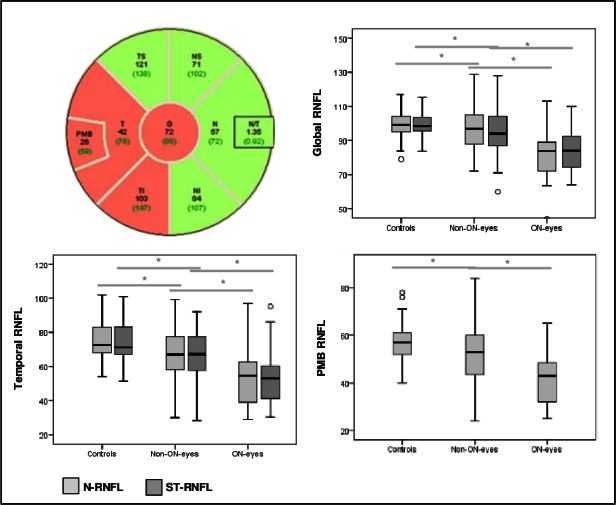
**Top left: Example of a peripapillary N-RNFL scan in an ON eye of a 23-year-old MS patient. RNFL thinning is present in the temporal sector and in the PMB (red: below normal limits, green: within normal limits). Top right and below: Boxplots of the global and temporal N-RNFL and ST-RNFL and the PMB N-RNFL values in controls, non-ON eyes, and ON eyes.** * indicates a *p*-value<0.05, generalized estimation equation (GEE) models. RNFL: retinal nerve fibre layer, N-RNFL: peripapillary scan technique with additional examination of the papillomacular bundle, ST-RNFL: standard peripapillary scan technique. G: global, PMB: papillo-macular bundle, T: temporal sector, TS: temporal superior sector, TI: temporal inferior sector, N: nasal sector, NS: nasal superior sector. NI: nasal inferior sector. Correction for multiple group comparisons was performed.

The mean values of the peripapillary RNFL thickness measured with N-RNFL and with ST-RNFL are displayed in Table 1 and Fig 1 separately for HC eyes and for MS eyes with and without previous ON. Overall, the mean global N-RNFL thickness and in the N-RNFL nasal inferior sector was lower in ON eyes than in non-ON eyes in all peripapillary sectors (p<0.05, GEE analysis Bonferroni correction to correct for multiple group comparisons; Table 1, Fig 1). Compared with HC eyes the RNFL-thickness was reduced not only in ON eyes but also in non-ON eyes in almost all peripapillary sectors for both measurement algorithms (p<0.05, GEE analysis with Bonferroni correction to correct for multiple group comparisons; Table 1, Fig 1). When correcting for multiple testing, significant differences between ON eyes and non-ON eyes were observed only for the global N-RNFL thickness and the N-RNFL in the nasal inferior sectors (S1 Table).

#### Differences between the RNFL thickness values obtained with N-RNFL and ST-RNFL

Comparison of the mean peripapillary RNFL thickness values in all eyes examined revealed no significant differences between the two scanning techniques, N-RNFL and ST-RNFL (Table 2).

**Table 2:** Mean peripapillary RNFL thickness values in all eyes examined (n = 308) across the different anatomical regions and comparison of the measurement obtained with both techniques (N-RNFL and ST-RNFL). There are no significant differences between the two scanning techniques.

	Mean (μm), SD	p-value*
N-RNFL-G	96.3±11.3	
ST-RNFL-G	95.3±11.7	p = 0.596
N-RNFL-T	68.5±15.5	
ST-RNFL-T	67.7±15.3	p = 0.693
N-RNFL-PMB	52.4±11.5	no comparison
N-RNFL-TI	140.1±24.5	
ST-RNFL-TI	139.7±24.0	p = 0.890
N-RNFL-TS	131.2±19.1	
ST-RNFL-TS	131.1±18.8	p = 0.983
N-RNFL-N	72.5±14.5	
ST-RNFL-N	71.9±13.3	p = 0.668
N-RNFL-NI	107.3±22.0	
ST-RNFL-NI	103.9±20.9	p = 0.205
N-RNFL-NS	105.4±22.7	
ST-RNFL-NS	105.7±21.9	p = 0.912

Abbreviations: SD: standard deviation, N-RNFL: peripapillary scan technique with additional examination of the papillomacular bundle, ST-RNFL: standard peripapillary scan technique. G: global, T: temporal sector, TS: temporal superior sector, TI: temporal inferior sector, N: nasal sector, NS: nasal superior sector, NI: nasal inferior sector.

* Mann-Whitney U test

#### Sensitivity and specificity of OCT (N-RNFL and ST-RNFL) to detect ON

**Fig 2:**
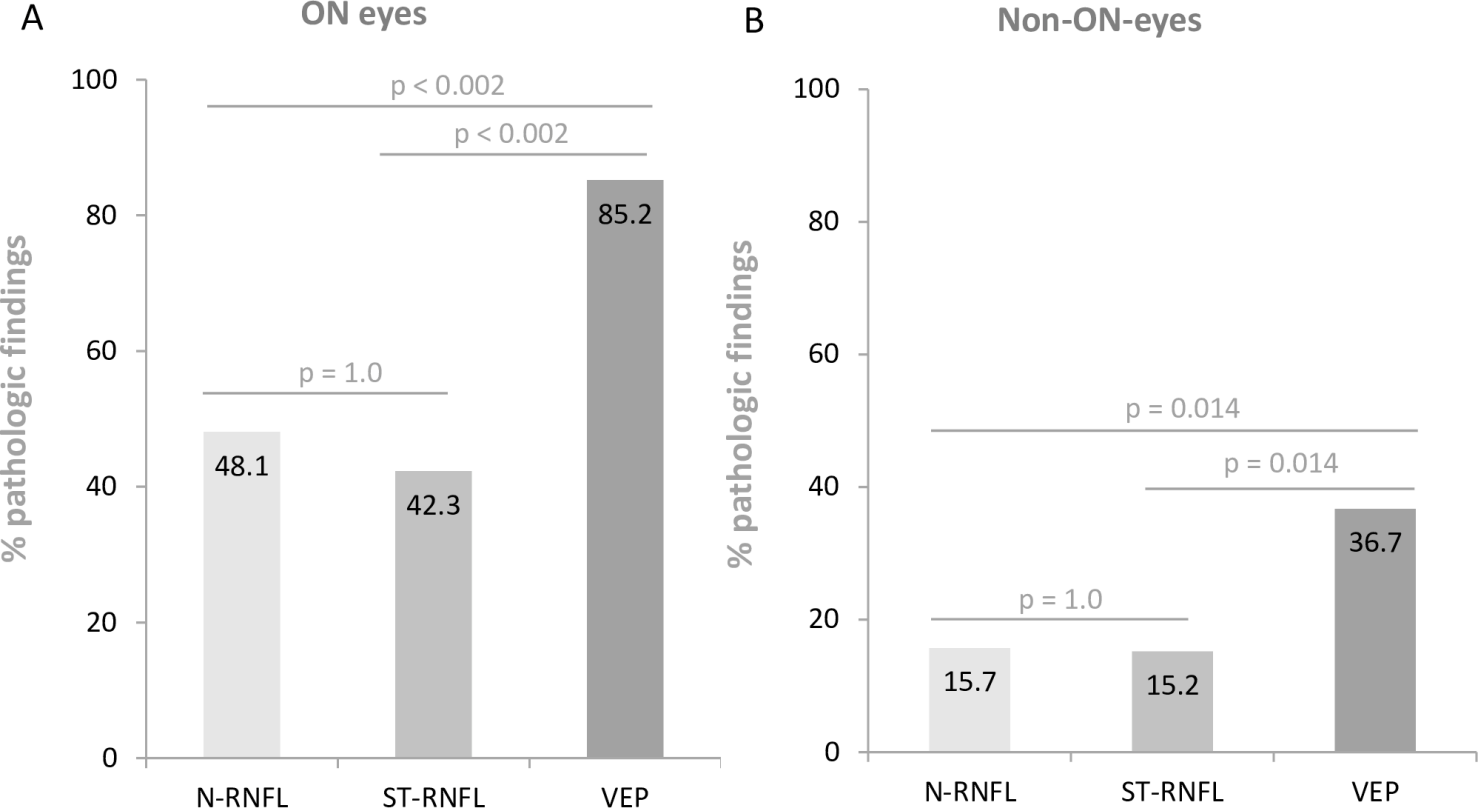
Comparison of the sensitivity of VEP, OCT, and their combination in all MS eyes with and without previous ON. **A: The sensitivity of VEP is superior to that of OCT (ST-RNFL and N-RNFL). B: Pathologic findings are more frequent in VEP than in OCT for non-ON eyes.** RNFL: retinal nerve fibre layer, N-RNFL: peripapillary scan technique with additional examination of the papillomacular bundle, ST-RNFL: standard peripapillary scan technique. ON: optical neuritis, VEP: visual evoked potentials.

Global RNFL thickness was abnormal in 15.1% of all MS eyes when assessed with the N-RNFL algorithm and in 13.7% with the ST-RNFL algorithm (McNemar test p = 1.0, Fig 2). VEPs were more frequently altered, with pathologic findings in 52.1% of all eyes.

These differences between the OCT and VEP results were significant (McNemar test N-RNFL vs VEP p<0.002; ST-RNFL vs VEP p<0.002).

**Fig 3:**
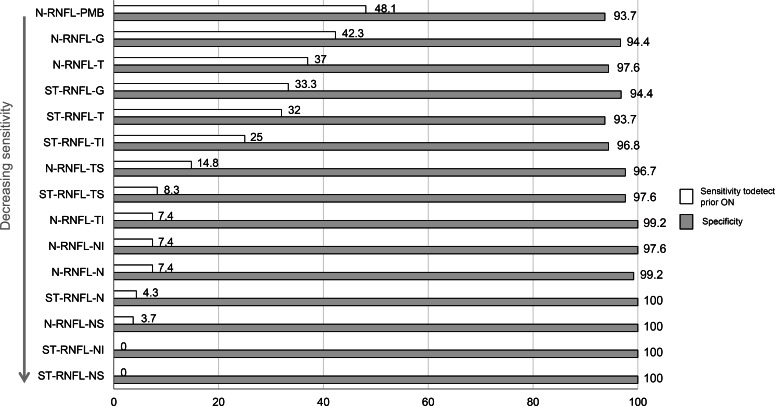
Sensitivity and specificity to detect ON in MS patients. Abbreviations: N-RNFL: N-sight retinal nerval fibre layer, ST-RNFL: standard retinal nerval fibre layer, PMB: papillo-macular bundle, T: temporal sector, TS: temporal superior sector, TI: temporal inferior sector, G: global, NI: nasal inferior sector, N: nasal sector, NS: nasal superior sector, TI: temporal inferior sector.

The sensitivity for previous ON was highest, at 48.1%, in the RNFL of the PMB. Global N-RNFL had sensitivity of 42.3% and specificity of 93.7%, while the corresponding figures for global ST-RNFL were 33.3% and 94.4%, respectively. These differences were not statistically significant (McNemar test PMB N-RNFL vs ST-RNFL-G p = 0.690) (Fig 3).

#### Sensitivity for identification of MS patients versus controls

**Fig 4:**
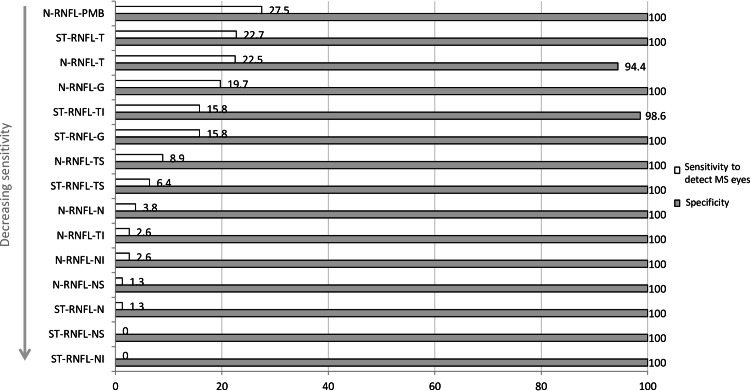
Sensitivity and specificity for detection of MS eyes (n = 73) within all eyes examined (n = 147). **The sensitivity is highest for the RNFL values within the PMB and the temporal sector on N-RNFL and ST-RNFL.** Abbreviations: N-RNFL: N-sight retinal nerval fibre layer, ST-RNFL: standard retinal nerval fibre layer, PMB: papillo-macular bundle, T: temporal sector, TS: temporal superior sector, TI: temporal inferior sector, G: global, NI: nasal inferior sector, N: nasal sector, NS: nasal superior sector, TI: temporal inferior sector.

The N-RNFL measurement in the PMB sector was abnormal in 27.5% (95% CI 18.1–37.5) of MS eyes, and the two methods had similar sensitivity in the temporal sector (22.7% (95% CI 12.0–30.7)/22.5% (95% CI 13.3–32.0)). The specificity of RNFL measures for MS was between 94.4% and 100% (Fig 4). VEP were pathologic in 85.2% (95% CI 70.0–96.7) of ON eyes in MS patients (McNemar test VEP versus global N-RNFL or ST-RNFL p<0.001, Fig 2).

In eyes of MS patients without a history of ON (Fig 2), ST-RNFL recordings were abnormal in 8/51 eyes (15.7% (95% CI 8.3–28.3)), and N-RNFL values in 8/52 eyes (15.4% (95% CI 10.2–33.3)) (McNemar Test p = 1.0); VEPs were abnormal in 36.7% (95% CI 23.5–51.8) (RNFL measures versus VEP, McNemar test p = 0.007). The combined VEP and OCT findings had sensitivity of 85.2% (95% CI 70.0–96.7) (for both global N-RNFL and ST-RNFL) for previous ON.

#### Agreement of OCT and VEP

**Fig 5:**
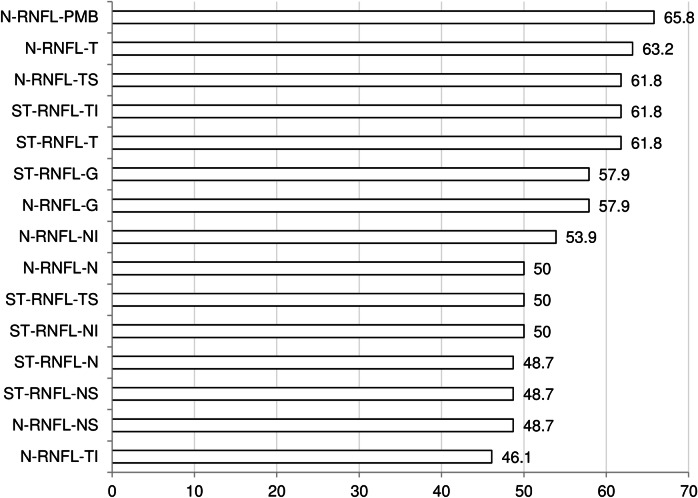
Agreement of OCT with pathologic VEP findings across all peripapillary sectors with N-RNFL and ST-RNFL. **The highest agreement of OCT and history of ON was for the values within the temporal sector in both N-RNFL and ST-RNFL.** Abbreviations: N-RNFL: N-sight retinal nerval fibre layer, ST-RNFL: standard retinal nerval fibre layer, PMB: papillo-macular bundle, T: temporal sector, TS: temporal superior sector, TI: temporal inferior sector, G: global, NI: nasal inferior sector, N: nasal sector, NS: nasal superior sector, TI: temporal inferior sector.

In general, the agreement of OCT and VEP findings was lower than the agreement of OCT with a history of ON. The best agreement of RNFL thickness and VEP findings was observed in the PMB (65.8%) and the temporal sector (N-RNFL 63.2%, ST-RNFL 61.8%; c.f. Fig 5).

### Regression analysis

In the GEE regression analysis, global RNFL thickness measured with method 1 (N-RNFL) and method 2 (ST-RNFL) was significantly associated with corrected VA, EDSS and disease duration (p<0.001/p = 0.006/p<0.001) in all eyes and in ON eyes (S1 Table). In all eyes, ON eyes, and non-ON eyes, temporal RNFL was associated with EDSS and disease duration, but not with corrected VA. In ON eyes, there was a strong association of RNFL thinning in the majority of peripapillary sectors (S2 Table). Again, it became obvious that PMB and temporal RNFL thicknesses were not associated with corrected VA.

## Discussion

We used sensitivity for a history of ON as a paradigm for the assessment of axonal loss in MS. The major findings of our study are the following: (1) We observed no significant change in the sensitivity for former ON when applying the N-RNFL protocol with the PMB as an additional sector. (2) OCT had lower sensitivity than VEP in post-ON eyes. (3) The combination of OCT and VEP did not increase sensitivity. (4) N-RNFL and ST-RNFL were correlated with corrected VA and EDSS in eyes without previous ON, and global N-RNFL correlated with corrected VA in all MS eyes. We observed no significant differences in the ability to detect previous clinically apparent ON between ST-RNFL and N-RNFL methods as determined by global and sectorial RNFL thickness. Although separate assessment of the PMB sector using N-RNFL depicted ON with a trend towards higher sensitivity (48.1%) compared with global measures from both measurement algorithms (42.3 and 33.3%), this difference was not statistically significant. This is in line with a former observation in patients with at least one former ON [14]. In this study, focusing on RNFL analysis in the temporal sector did not increase the sensitivity for ON. However, the study did not apply the N-RNFL protocol and therefore, cannot comment on isolated PMB RNFL values.

A difficulty in validation studies for the use of OCT to quantify retinal pathology in MS is the lack of a gold standard. We compared two measurement techniques to match findings with VEP in eyes with and without a history of previous ON. A previous OCT study reported abnormal RNFL thickness in 68% and pathologic VEP in 86% of ON eyes [15]. While our VEP findings were very similar (85.2%) the maximum sensitivity of both OCT techniques applied in our study was only 48.1% [15]. In contrast to findings from the same study, in our hands the combination of OCT and VEP did not increase the sensitivity for previous ON in MS patients (83.3% compared to 89.3%, reported previously [15]).

Our findings showed a superior sensitivity of VEP for previous ON. This is in line with a lower sensitivity of OCT (60%) than VEP (81%) observed in 65 subjects with at least one clinical ON episode and a diagnosis of MS, neuromyelitis optica, CIS, or idiopathic ON [14] and also corroborates the sensitivity of 68% for OCT and 86% for VEP to former ON as described recently [15].

In accordance with independent observations from the studies mentioned above [14,15], we found that OCT detected fewer optic nerve changes in the clinically unaffected eye than VEP (8.2–15.4% versus 36.9%). Other studies stated similar (6%) or higher rates (19.2–35.7%) of pathologic OCT measurements in non-ON eyes [14,16,17]. The comparability of these results is limited by the different technical devices used [18], the varying characteristics of study participants (e.g., neurological diagnosis, disease duration, number of previous ON) and the differing sample sizes. The reduction of RNFL thickness in the absence of a history of clinically evident ON has been attributed to subclinical ON or, alternatively, to retrograde trans-synaptic degeneration after retrogeniculate lesions or subtle chronic demyelination and axonal loss [3,19–25] in MS. We observed significantly lower RNFL thickness in non-affected MS eyes than in HC eyes, in line with previous reports [26–29]. Furthermore, the good correlation of RNFL, EDSS, and disease duration in non-ON eyes documented in this study parallels previous findings [14] and supports the notion that non-ON eyes are more suitable for the monitoring of axonal damage, apart from clinical relapses [29].

This highlights the value of OCT for assessment of neuronal loss as a surrogate for progression of disability. Our study has strengths and limitations. While patients were recruited prospectively and their data were compared to the findings in an age- and sex-matched control group, our study was only cross-sectional and we have no follow-up data. This might be important when comparing VEP to RFNL results, as it has been proposed that prolongations of VEP latencies may diminish for at least 2 years after ON [30]. Moreover, we did not perform perimetry, which should be included in further studies because, in some cases, ON may cause exclusively peripheral visual field defects which are often not detected by full-field VEP [30].

Our study shows that the two segmentation techniques in SD-OCT, ST-RNFL and N-RNFL, are of similar value for distinction between ON eyes and non-ON eyes in MS patients and healthy controls. We conclude that both algorithms are adequate for examining the peripapillary RNFL of MS patients. This has direct practical implications because ST-RNFL is widely used among general ophthalmologists. The N-RNFL protocol may serve as an additional useful tool for special issues. Overall, the sensitivity of OCT for the detection of ON was substantially lower than that of VEP. Due to this fact, VEP remains the most important diagnostic tool to screen for past or subclinical ON.

## Supporting Information

S1 Dataset (XLSX)

S1 Table Bonferroni-corrected RNFL thickness values in controls and MS-patients in all peripapillary sectors and comparison among groups. RNFL: retinal nerve fibre layer, N-RNFL: peripapillary scan technique with additional examination of the papillomacular bundle, ST-RNFL: standard peripapillary scan technique, SD: standard deviation. G: global, PMB: papillo-macular bundle, T: temporal sector, TS: temporal superior sector, TI: temporal inferior sector, N nasal sector, NS: nasal superior sector, NI: nasal inferior sector. p-values below the level of significance of 0.05 are bold.
(DOC)

S2 Table Association between pathologic global/sectorial N-RNFL/ST-RNFL measurements and visual acuity (VA), EDSS (Expanded disability Status Scale) and Disease duration in all eyes, ON-eyes and non-ON-eyes (generalized estimation equations). Abbreviations: Visual acuity (VA), EDSS (Expanded disability Status Scale); “-”indicates that there were no pathologic RNFL-findings in the subgroup of patients within this sector. B = correlation coefficient Beta. RNFL-N: method 1, examination of 7 peripapillary sectors, RNFL-M: method 2, examination of 6 peripapillary sectors. G: global, PMB: papillo-macular bundle, T: temporal sector, TS: temporal superior sector, TI: temporal inferior sector, N: nasal sector; NI: nasal inferior sector, nasal sector; NS: nasal superior sector, TI: temporal inferior sector. p-values below the level of significance of 0.05 are bold.
(DOC)
