# Supplementary material for: Adding Papillomacular Bundle Measurements to Standard Optical Coherence Tomography Does Not Increase Sensitivity to Detect Prior Optic Neuritis in Patients with Multiple Sclerosis
Source: PLoS One. 2016 May 12;11(5):e0155322. doi: 10.1371/journal.pone.0155322 (PMC4865166; doi:10.1371/journal.pone.0155322)
Supplement: S1 Table — RNFL: retinal nerve fibre layer, N-RNFL: peripapillary scan technique with additional examination of the papillomacular bundle, ST-RNFL: standard peripapillary scan technique, SD: standard deviation. G: global, PMB: papillo-macular bundle, T: temporal sector, TS: temporal superior sector, TI: temporal inferior sector, N nasal sector, NS: nasal superior sector, NI: nasal inferior sector. p-values below the level of significance of 0.05 are bold. (DOC) [file pone.0155322.s002.doc]

|  | **Thickness, µm, mean value, SD** | | | **p-values** | | |
| --- | --- | --- | --- | --- | --- | --- |
|  | **Controls (n=74 eyes)** | **Non-ON eyes (n=53)** | **ON-eyes (n=24)** | **Non-ON-eyes versus controls** | **Non-ON versus ON-eyes** | **ON-eyes versus controls** |
| **N-RNFL** |  |  |  |  |  |  |
| N-RNFL-G | 99.4±8.6 | 98.3±14.2 | 87.3±31.7 | **p<0.007** | **p=0.035** | **p=0.007** |
| N-RNFL-PMB | 56.9±7.71 | 51.1±11.7 | 43.9±12.9 | **p<0.007** | p=0.119 | **p=0.007** |
| N-RNFL-T | 74.6±11.2 | 66.5±14.6 | 57.4±17.8 | **p<0.007** | p=0.098 | **p=0.014** |
| N-RNFL-TS | 136.1±13.2 | 135.3±26.7 | 121.2±26.5 | **p<0.007** | p=0.224 | p=0.056 |
| N-RNFL-TI | 146.8±18.9 | 139.4±29.0 | 139.7±30.1 | **p<0.007** | p=0.322 | **p=0.028** |
| N-RNFL-N | 74.6±14.4 | 74.4±17.4 | 75.3±33.7 | p=0.357 | p=0.490 | p=0.112 |
| N-RNFL-NS | 106.5±23.4 | 107.1±25.6 | 100.9±38.2 | **p=0.021** | p=0.077 | **p=0.014** |
| N-RNFL-NI | 108.1±20.9 | 113.3±24.5 | 102.8±30.5 | **p=0.007** | **p=0.049** | **p=0.021** |
| **ST-RNFL** |  |  |  |  |  |  |
| ST-RNFL-G | 99.4±7.2 | 94.0±14.2 | 87.8±17.8 | **p<0.006** | p=0.090 | **p=0.012** |
| ST-RNFL-T | 73.9±11.3 | 67.1±16.6 | 58.2±21.6 | **p<0.006** | p=0.078 | **p=0.030** |
| ST-RNFL-TS | 136.6±12.6 | 132.5±27.9 | 121.1±21.0 | **p=0.036** | p=0.684 | p=0.444 |
| ST-RNFL-TI | 145.9±18.6 | 140.7±29.1 | 127.0±23.1 | **p=0.006** | p=0.294 | p=0.066 |
| ST-RNFL-N | 74.3±13.5 | 72.8±14.9 | 67.0±22.6 | p=0.324 | p=0.756 | p=0.102 |
| ST-RNFL-NS | 107.5±23.6 | 108.7±19.9 | 99.4±42.5 | p=0.144 | p=0.312 | **p=0.042** |
| ST-RNFL-NI | 105.3±19.9 | 108.2±23.1 | 92.0±29.5 | **p=0.030** | p=0.246 | p=0.072 |

**S1 Table. Bonferroni-corrected RNFL thickness values in controls and MS-patients in all peripapillary sectors and comparison among groups.**

RNFL: retinal nerve fibre layer, N-RNFL: peripapillary scan technique with additional examination of the papillomacular bundle, ST-RNFL: standard peripapillary scan technique, SD: standard deviation. G: global, PMB: papillo-macular bundle, T: temporal sector, TS: temporal superior sector, TI: temporal inferior sector, N nasal sector, NS: nasal superior sector, NI: nasal inferior sector. p-values below the level of significance of 0.05 are bold.
