# Supplementary material for: Adding Papillomacular Bundle Measurements to Standard Optical Coherence Tomography Does Not Increase Sensitivity to Detect Prior Optic Neuritis in Patients with Multiple Sclerosis
Source: PLoS One. 2016 May 12;11(5):e0155322. doi: 10.1371/journal.pone.0155322 (PMC4865166; doi:10.1371/journal.pone.0155322)
Supplement: S2 Table — Abbreviations: Visual acuity (VA), EDSS (Expanded disability Status Scale); “-”indicates that there were no pathologic RNFL-findings in the subgroup of patients within this sector. B = correlation coefficient Beta. RNFL-N: method 1, examination of 7 peripapillary sectors, RNFL-M: method 2, examination of 6 peripapillary sectors. G: global, PMB: papillo-macular bundle, T: temporal sector, TS: temporal superior sector, TI: temporal inferior sector, N: nasal sector; NI: nasal inferior sector, nasal sector; NS: nasal superior sector, TI: temporal inferior sector. p-values below the level of significance of 0.05 are bold. (DOC) [file pone.0155322.s003.doc]

|  | **VA** | **EDSS** | **Disease duration** |
| --- | --- | --- | --- |
| **All eyes (n=73)** | | | |
| N-RNFL-G | **B=0.004 p<0.001** | **B=-0.058 p=0.006** | **B=-1.709 p=0<001** |
| ST-RNFL-G | **B=0.003 p=0.001** | B=1.683 p=0.098 | **B=-1.406 p=0.001** |
| N-RNFL-T | B=0.001 p=0.161 | **B=-0.059 p<0.001** | **B=-0.917 p=0.029** |
| ST-RNFL-T | B=0.001 p=0.141 | **B=-0.058 p=0.001** | **B=-0.917 p=0.021** |
| **ON eyes (n=24)** | | | |
| N-RNFL-G | **B=0.004 p=0<001** | **B=-0.058 p=0.006** | **B=1.709 p<0.001** |
| ST-RNFL-G | B=0.001 p=0.141 | **B=-0.055 p=0.012** | **B=-1.406 p=0.001** |
| N-RNFL-T | B=0.001 p=0.161 | **B=-0.059 p<0.001** | **B=-0.971 p=0.029** |
| ST-RNFL-T | B=0.001 p=0.141 | **B=-0.058 p=0.001** | **B=-0.971 p=0.021** |
| N-RNFL-TI | **B=0.001 p<0.022** | **B=-0.027 p=0.014** | **B=-0.693 p=0.006** |
| ST-RNFL-TI | **B=0.002 p<0.001** | **B=-0.024 p=0.021** | **B=-0.701 p=0.001** |
| N-RNFL-TS | **B=0.002 p=0.028** | **B=-0.039 p=0.005** | **B=-1.041 p<0.001** |
| ST-RNFL-TS | **B=0.002 p=0.023** | **B=-0.036 p=0.018** | **B=-1.175 p<0.001** |
| N-RNFL-N | **B=0.004 p<0.001** | **B=-0.048, p<0.005** | **B=-1.568 p=0.001** |
| ST-RNFL-N | **B=0.004 p<0.001** | **B=-0.043 p=0.007** | **B=-1.521 p=0.001** |
| N-RNFL-NS | **B=0.003 p<0.001** | **B=-0.055 p<0.001** | **B=1.330 p<0.001** |
| ST-RNFL-NS | **B=0.004 p<0.001** | **B=-0.036 p=0.035** | **B=-0.026 p=0.008** |
| N-RNFL-NI | **B=0.004 p<0.001** | **B=-0.026 p=0.008** | **B=0.426 p=0.474** |
| ST-RNFL-NI | **B=0.004 p<0.001** | **B=-0.019 p=0.049** | B=-0.592 p=0.156 |
| N-RNFL-PMB | B=0.003 p=0.165 | **B=-0.110 p<0.001** | **B=-2.021 p=0.003** |
| **Non-ON eyes (n=49)** | | | |
| N-RNFL-G | **B=0.009 p=0<0.001** | **B=-0.058 p=0.006** | **B=1.954 p<0.001** |
| ST-RNFL-G | **B=0.009 p=0<0.001** | **B=-0.058 p<0.007** | **B=1.967 p<0.001** |
| N-RNFL-T | B=-0.001 p=0.812 | B=-0.025 p=0.458 | **B=2.830 p<0.001** |
| ST-RNFL-T | B<0.001 p=0.983 | B=-0.020 p=0.593 | **B=3.258 p<0.001** |
| N-RNFL-PMB | B=-0.007 p=0.453 | B=-0.014 p=0.841 | **B=5.747 p<0.001** |

**S2 Table. Association between pathologic global/sectorial N-RNFL/ST-RNFL measurements and visual acuity (VA), EDSS (Expanded disability Status Scale) and Disease duration in all eyes, ON-eyes and non-ON-eyes (generalized estimation equations).**

Abbreviations: Visual acuity (VA), EDSS (Expanded disability Status Scale); “-“ indicates that there were no pathologic RNFL-findings in the subgroup of patients within this sector.

B=correlation coefficient Beta. RNFL-N: method 1, examination of 7 peripapillary sectors, RNFL-M: method 2, examination of 6 peripapillary sectors. G: global, PMB: papillo-macular bundle, T: temporal sector, TS: temporal superior sector, TI: temporal inferior sector, N: nasal sector; NI: nasal inferior sector, nasal sector; NS: nasal superior sector, TI: temporal inferior sector. p-values below the level of significance of 0.05 are bold.
